# LncRNA LUCAT1 as a prognostic biomarker in cholangiocarcinoma through targeting miR-141-3p: clinical and functional insights

**DOI:** 10.1186/s41065-025-00512-6

**Published:** 2025-07-26

**Authors:** Yuxin An, Qing Chen, Shanshan Zhou, Chengcheng Ying, Guanbao Long, Zouxiao Hu, Jiangyang Sun, Niu Zhang

**Affiliations:** 1 School of Medicine, Jiangsu University, Zhenjiang, 212013 China; 2 Orthopedic of Department, Shenzhen Third People’s Hospital, Shenzhen, Guangdong Province, 518112 China; 3 Department of Emergency, Tianyou Hospital Affiliated to Wuhan University of Science and Technology, Wuhan, 430000 China; 4 Department of Urology, The Central Hospital of Wuhan, Tongji Medical College, Huazhong University of Science and Technology, Wuhan, 430014 China; 5 Department of Hepatobiliary Surgery, The Central Hospital of Wuhan, Tongji Medical College, Huazhong University of Science and Technology, Wuhan, 430014 China; 6 Department of Oncology, Zhenjiang Medical School of Nanjing Medical University, No. 8, Electric Power Road, Zhenjiang, Jiangsu, 212002 China

**Keywords:** Cholangiocarcinoma, Lung cancer-related transcript 1, Diagnosis, Prognosis

## Abstract

**Background:**

Cholangiocarcinoma (CHOL) has a poor prognosis due to its asymptomatic progression, challenges in early detection, and limited treatment options. The lncRNA LUCAT1 is highly expressed in several cancers, including lung, gastric, ovarian, and osteosarcoma tissues.

**Aim:**

This study investigates the potential of LUCAT1 as a diagnostic and prognostic biomarker for CHOL.

**Materials and methods:**

In this study, we collected tumor tissues and adjacent tumor healthy tissues from 83 CHOL patients. LUCAT1 expression was quantified in CHOL tissues and cell lines via RT-qPCR. Diagnostic and prognostic significance was assessed through ROC curves, Kaplan-Meier survival analysis, and Cox regression models. The biological effects of LUCAT1 on cell proliferation and migration were examined using QBC939 and HuCCT1 cells with transfection assays. The regulatory interaction between LUCAT1 and miR-141-3p was validated using a dual-luciferase reporter assay.

**Results:**

Elevated expression of LUCAT1 was observed in CHOL tumor tissues and human cholangiocarcinoma cells, correlating with tumor size, CA-19-9 levels, and TNM stage. The ROC curve, with an AUC of 0.908 (*p* < 0.001), effectively distinguished CHOL tumor tissues from adjacent non-tumor tissues. And its sensitivity and specificity in distinguishing CHOL tissues from normal tissues were 88.5% and 89.2%, respectively. Survival analyses linked LUCAT1 overexpression to poorer patient outcomes. Silencing LUCAT1 impaired the proliferation and migration of QBC939 and HuCCT1 cells. Dual-luciferase assay confirmed the regulatory relationship between miR-141-3p and LUCAT1. Inhibition of miR-141-3p reversed the effect of LUCAT1 on the proliferation and migration of QBC939 and HuCCT1 cells.

**Conclusion:**

LUCAT1 demonstrates significant diagnostic and prognostic potential and could serve as a novel biomarker for CHOL.

## Introduction

Cholangiocarcinoma (CHOL) is the most common primary malignancy of the biliary tract [1, 2]. CHOL can develop at any location within the biliary tract [3, 4]. CHOL is classified as extrahepatic or intrahepatic, with the second-order bile ducts serving as the anatomical boundary [5]. The prevalence and mortality of CHOL have been steadily increasing globally, with a particularly high burden in East Asia [6]. The risk of CHO is closely associated with cholelithiasis, chronic cholangitis, primary sclerosing cholangitis (PSC), liver fluke infection and genetic susceptibility [7–9]. At present, the treatment strategies for CHOL show a multidisciplinary integration feature: the main treatment methods include surgical treatment, chemotherapy and radiotherapy, combined immunotherapy, molecular targeted therapy, traditional Chinese medicine treatment etc [10–12]. CHOL is marked by latency, early aggressiveness, distant metastasis, and poor prognosis, posing significant challenges for its diagnosis and treatment [13, 14]. Surgery remains the primary curative option but is feasible only in early-stage disease [15]. Unfortunately, most patients are diagnosed at advanced stages, where the prognosis is poor, with a median survival of only 24 months [15]. Identifying biomarkers for CHOL is crucial to improving prognosis and developing novel therapies to prevent metastasis following radical hepatectomy.

Long noncoding RNAs (lncRNAs) have little to no protein-coding potential [16–18]. Studies have shown that lncRNAs regulate cellular processes such as development, growth, apoptosis, migration, and invasion [19, 20]. Certain lncRNAs have been found to play critical regulatory roles in cancers, including lung, gastric, colorectal, glioma, osteosarcoma, and CHOL [21, 22]. LUCAT1 has been implicated in the progression of several cancers, including gastric, lung, and ovarian malignancies, by modulating various miRNA-mediated pathways: it inhibits ferroptosis in bladder cancer by regulating STAT3 [23], suppresses glioblastoma growth when silenced [24], promotes gastric cancer proliferation and migration via the miR-134-5p/YWHAZ [25], and enhances metastasis in lung adenocarcinoma l [26]. However, the clinical diagnostic and prognostic significance of LUCAT1 in CHOL remains unexplored.

This study aims to explore the potential of LUCAT1 as a novel biomarker for early detection and prognostic prediction of CHOL, while also examining its interaction with miR-141-3p.

## Material and method

### Patients and tissue samples

Eighty-three CHOL patients who underwent surgery at The Central Hospital of Wuhan, Tongji Medical College, Huazhong University of Science and Technology, between February 2017 and March 2019, were included in this study. Post-surgical CHOL tissue specimens were preserved at -80 °C for RNA extraction, while adjacent tissues were confirmed as normal liver samples. Patients underwent postoperative follow-ups every three months for up to five years or until death. Informed consent was obtained from all participants prior to tissue collection, and the study was approved by the Ethics Committee of The Central Hospital of Wuhan, Tongji Medical College, Huazhong University of Science and Technology (2018016).

### Cell culture and transfection

Cholangiocarcinoma cell lines QBC939, HuCCT1, CCLP1, QBL, and human intrahepatic bile duct epithelial cells (hIBEpiC, ATCC) were cultured in DMEM (Gibco) supplemented with 10% FBS under standard conditions of 37 °C, 5% CO_2,_ and 95% air. 5 µg/µL LUCAT1-siRNA and control plasmids (ThermoFisher, USA), 5 µg/µL miR-NC, miR-141-3p mimics, and miR-141-3p inhibitors (RIB BIO, Guangzhou, China) were transfected into QBC939 and HuCCT1 cells using Lipofectamine 3000 (ThermoFisher, USA).

### Cell proliferation

Logarithmically growing cells were seeded into 96-well plates, and proliferation was assessed using the CCK-8 kit (mlbio, Shanghai, China) at 0, 24, 48, and 72 h. After adding 10 µL of CCK-8 reagent to each well, cells were incubated at 37 °C for 2 h, and OD values were recorded at 450 nm.

### Cell migration

Cells were resuspended in serum-free medium containing BSA and adjusted to a density of 5 × 10⁵/ml. A 100 µL suspension was seeded in the upper chamber of transwells, while the lower chamber contained medium with 20% serum. After 48 h of incubation at 37 °C, cells were fixed, stained, and counted in five randomly selected fields of view.

### RT-qPCR

Total RNA was extracted from 83 paired frozen CHOL specimens and cultured cells using the TRIzol Kit (ThermoFisher, USA) and reverse-transcribed into cDNA using the RNeasy Mini Kit (Qiagen). GAPDH and U6 served as internal controls for quantifying LUCAT1 and miR-141-3p expression, respectively. Relative expression levels were calculated using the 2^−ΔΔCt^ method normalized to GAPDH (LUCAT1) and U6 (miR-141-3p). Standard curves were established for relative quantification using GAPDH and U6 as internal controls. Below are the primer sequences: LUCAT1, Forward 5’- GCTCGGATTGCCTTAGACAG − 3’ and Reverse 5’ - GGGTGAGCTTCTTGTGAGGA − 3’; GAPDH, Forward 5’ - CTCCAGTACCTACCTTACAGGGATT − 3’ and Reverse 5’ - GCTGCTGGCACCTCCA − 3’; miR-141-3p, Forward 5’ - GAATCCCGAACTGCTCA − 3’ Reverse 5’ - AAGGTGAAGTGGTAGCAAA − 3’; U6, Forward 5’ - CTCGCTTCGGCAGCACA − 3’ and Reverse 5’ - AACGCTTCAGGAATTTGCGT − 3’.

### Luciferase reporter assay

QBC939 and HuCCT1 cells were seeded into 96-well plates (37 °C, 5% CO2) and co-transfected with pmirGLO-LUCAT1-WT/MUT reporter plasmids, NC mimics, miR-141-3p mimics, and inhibitors. After 24 h, cells were lysed using Sigma lysis buffer, and luciferase activity was measured with ONE-Glo™ EX Luciferase Assay Reagent (Promega) on a Synergy HTX plate reader (BioTek). Results were normalized to Renilla luciferase activity.

### Statistical analysis

Data were expressed as mean ± SD. Statistical analyses included unpaired t-tests, chi-square tests, and one-way ANOVA. The diagnostic utility of LUCAT1 in CHOL was evaluated using ROC analysis, while its prognostic significance was assessed via Cox regression and Kaplan-Meier analyses. A p-value < 0.05 was considered statistically significant. For all experiments, three parallel trials were performed.

## Results

### LUCAT1 expression was significantly upregulated in CHOL tumor tissues and cell lines

**Fig. 1:**
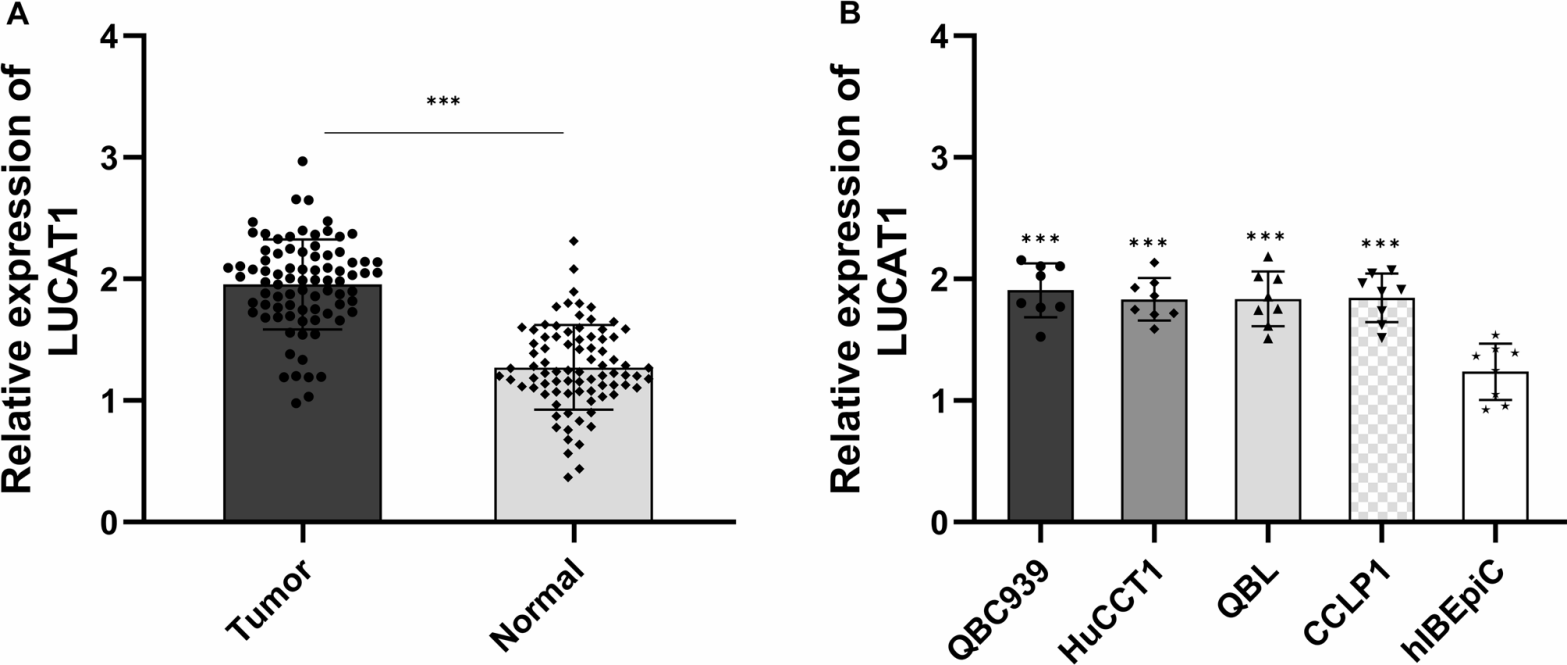
LUCAT1 expression levels were upregulated in CHOL tumor tissues and cell lines. **(A)** The LUCAT1 expression in cholangiocarcinoma tumors and normal tissues was tested by RT-qPCR. Compared with normal tissue, LUCAT1 was upregulated in cholangiocarcinoma tumor tissues. **(B)** The LUCAT1 expression in QBC939, HuCCT1, CCLP1, QBL, and hIBEpiC cells was tested by RT-qPCR. Compared with normal bile duct epithelial cells, LUCAT1 was upregulated in cholangiocarcinoma tumor cell lines. ***P < 0.001. All experiments were performed more than three times and statistical tests were performed using paired t-test and ordinary one-way ANOVA

LUCAT1 expression was assessed in paired tumor and normal specimens using RT-qPCR, revealing significant upregulation in CHOL tumor tissues (*p* < 0.001, Fig. 1A). To investigate whether LUCAT1 acts as a potential oncogene in CHOL, its expression was analyzed in four CHOL cell lines (QBC939, HuCCT1, CCLP1, and QBL) via RT-qPCR. LUCAT1 expression was significantly higher in CHOL cell lines compared to hIBEpiC controls (*p* < 0.001, Fig. 1B).

### Relationship of LUCAT1 with CHOL patients’ clinicopathological features

The correlation between clinicopathological features and LUCAT1 expression in CHOL patients was evaluated based on the average expression levels in tissue specimens. Age, gender, tumor number, and primary tumor location showed no significant correlation with LUCAT1 expression levels (*p* = 0.729 with age, *p* = 0.51 with gender, *p* = 0.386 with tumor number, *p* = 0.422 with primary tumor location, Table 1). Tumor size, CA-19-9 levels, and TNM-T stage were significantly associated with LUCAT1 expression (*p* = 0.034 for tumor size, *p* < 0.001 for CA-19-9, *p* = 0.012 for TNM-T stage; Table 1), suggesting their involvement in CHOL progression.

**Table 1:** Association between CHOL patients’ clinicopathological features and LUCAT1 expression levels

Variable	Total	LUCAT1 expression level		
	*N* = 83	High = 47	Low = 36	*p*-value
Age				
>60	42	23	19	0.729
≤ 60	41	24	17	
Gender				
Female	38	23	15	0.51
Male	45	24	21	
Tumor Size				
>5	41	28	13	0.034
≤ 5	42	19	23	
Tumor Number				
Single	68	37	31	0.386
Multiple	15	10	5	
CA-19-9, U/mL				
>37	47	38	9	<0.001
≤ 37	36	9	27	
Primary tumor location				
Intrahepatic	72	42	30	0.422
Extrahepatic and perihilar	11	5	6	
TNM-T-Stage				
T1	40	17	23	0.012
T2-T3	43	30	13	

### Diagnostic and prognostic value of LUCAT1 in CHOL

**Fig. 2:**
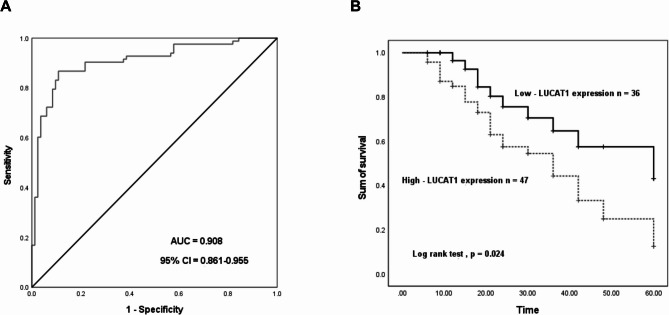
Diagnostic and prognostic value of LUCAT1 in CHOL. **(A)** LUCAT1 could distinguish cholangiocarcinoma tumor tissues from normal tissues with an AUC of 0.908. **(B)** Cholangiocarcinoma patients with high-LUCAT1 expression have a poor prognosis

ROC analysis demonstrated the significant diagnostic capability of LUCAT1 in distinguishing CHOL tumor tissues from normal tissues, with an AUC of 0.908 (*p* < 0.001, Fig. 2A). The ROC curve showed sensitivity and specificity values of 88.5% and 89.2%, respectively (Fig. 2A). CHOL patients were categorized into high- and low-LUCAT1 expression groups based on the average expression levels in tissue specimens. Kaplan-Meier analysis revealed that patients with low LUCAT1 expression exhibited better prognosis compared to those with high expression (log-rank test, *p* = 0.024, Fig. 2B). Multivariate Cox regression analysis confirmed that LUCAT1 serves as an independent prognostic indicator for CHOL (HR = 2.484, *p* = 0.035, Table 2).

**Table 2:** Multivariate Cox regression analysis evaluating the prognostic value of CHOL patients’ clinicopathological features and LUCAT1

	HR factor	(95% CI)	*p*
LUCAT1	2.484	1.064–5.797	0.035
Age	1.319	0.641–2.713	0.452
Gender	1.089	0.535–2.217	0.813
Tumor Size	2.189	0.999–4.796	0.050
Tumor Number	1.429	0.558–3.659	0.273
CA-19-9, U/mL	1.523	0.762–3.043	0.233
Primary tumor location	2.118	0.885–5.065	0.092
TNM-T-Stage	2.237	0.641–2.713	0.068

### Effects of LUCAT1 on CHOL tumor cells

**Fig. 3:**
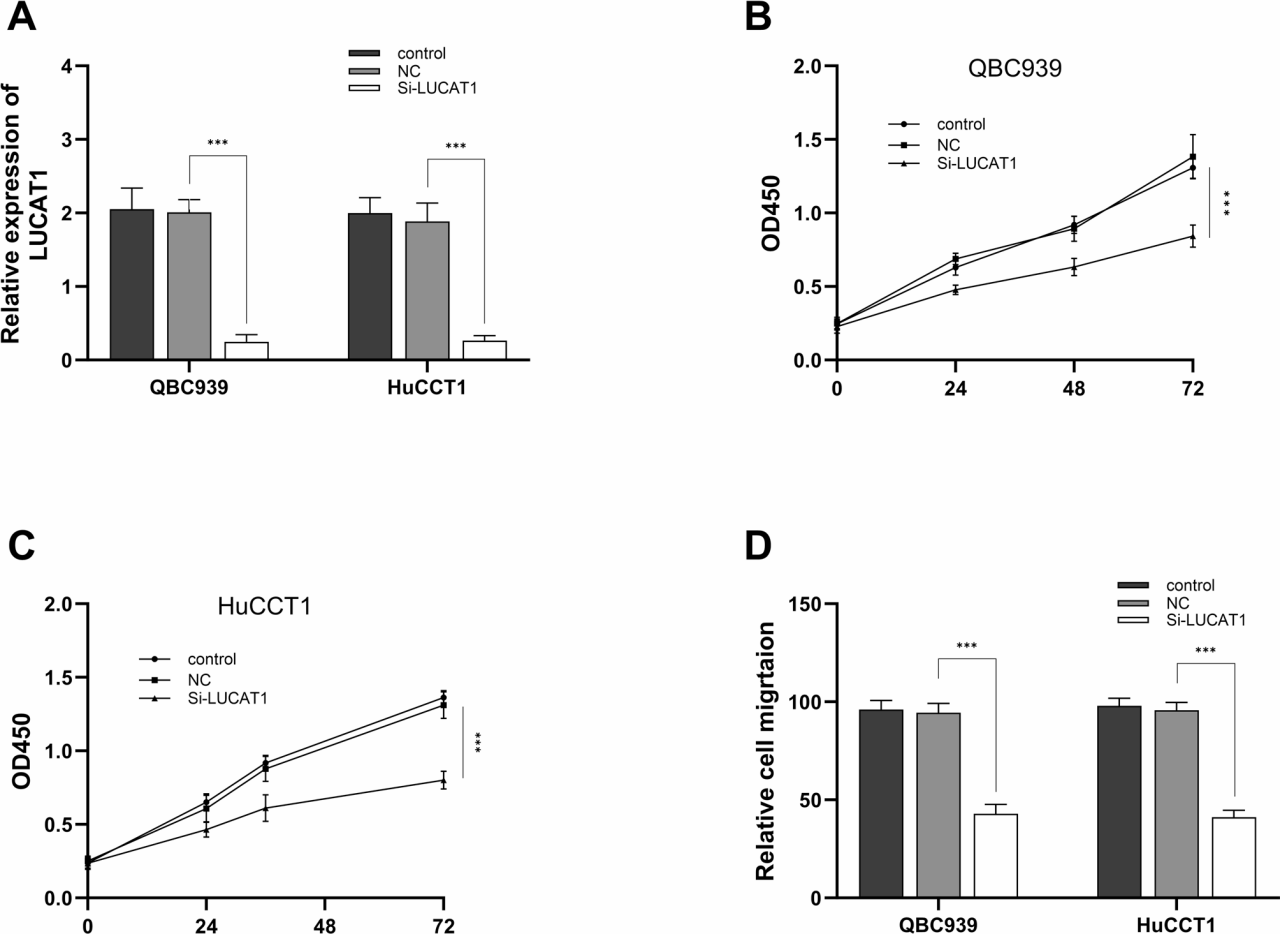
Interaction of LUCAT1 and miR-141-3p. **(A)** The LUCAT1 expression in QBC939 and HuCCT1 was detected by RT-qPCR. QBC939 and HuCCT1 cells were successfully transfected with si-LUCAT1 using Lipofectamine 3000. Effects of silencing LUCAT1 on QBC939 **(B)** and HuCCT1 **(C)** cell proliferation. **(D)** Effects of silencing LUCAT1 on QBC939 and HuCCT1 on cell migration. *** *P* < 0.001. All experiments were performed more than three times and statistical tests were performed ordinary two-way ANOVA

QBC939 and HuCCT1 cells were effectively transfected with si-LUCAT1 using Lipofectamine 3000 (*p* < 0.001, Fig. 3A). Next, we explored the effects of LUCAT1 on cell proliferation and migration of the human cholangiocarcinoma cells QBC939 and HuCCT1. We examined the growth of QBC939 and HuCCT1 cells at 24, 48, and 72 h by CCK-8 method. We found that silencing LUCAT1 inhibited cell proliferation of QBC939 and HuCCT1cells (*p* < 0.001, Fig. 3B-C). Meanwhile, the cell migration assay result showed that silencing LUCAT1 inhibited the migration of QBC939 and HuCCT1 cells. (*p* < 0.001, Fig. 3D).

### LUCAT1 targets miR-141-3p to affect CHOL tumor cells

**Fig. 4:**
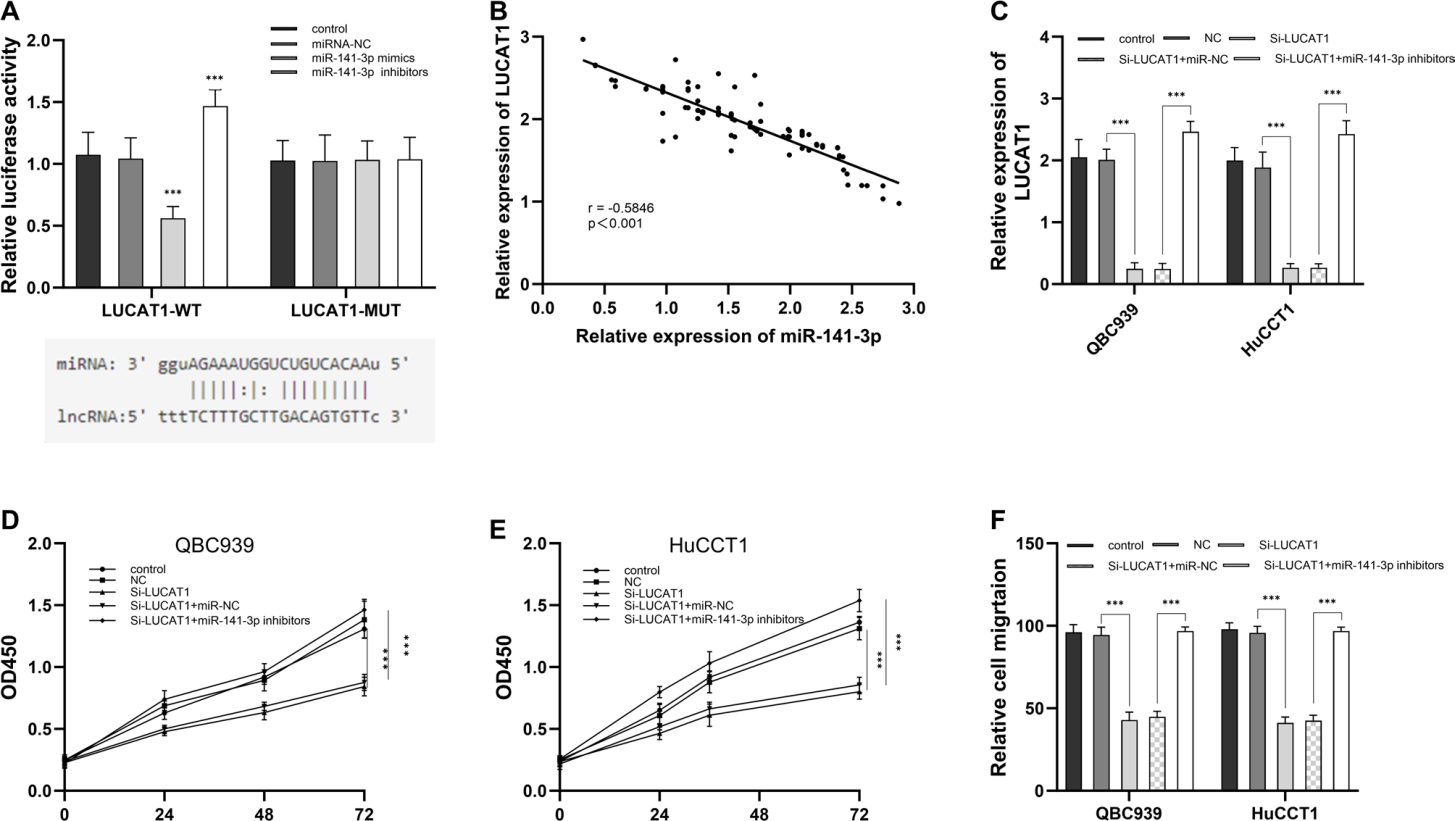
LUCAT1 targets miR-141-3p to affect CHOL tumor cells. **(A)** miR-141-3p mimics inhibited the luciferin activity of LUCAT1-WT, which was increased by miR-141-3p inhibitors. **(B)** The LUCAT1 expression was negatively correlated with the miR-141-3p expression in CHOL tumor tissue with *r* = -0.5846, *p* < 0.001. **(C)** The LUCAT1 expression in QBC939 and HuCCT1 was detected by RT-qPCR. QBC939 and HuCCT1 cells were successfully transfected with si-LUCAT1 and si-LUCAT1 + miR-141-3p using Lipofectamine 3000. miR-141-3p reversed the inhibitory effect of silencing LUCAT1 on the proliferation of QBC939 **(D)** and HuCCT1 **(E)** cells. **(F)** miR-141-3p reversed the inhibitory effect of silencing LUCAT1 on the cell migration of QBC939 and HuCCT1 cells. *** *P* < 0.001. All experiments were performed more than three times and statistical tests were performed ordinary two-way ANOVA

The dual-luciferase reporter assay revealed that the miR-141-3p mimics inhibited the luciferase activity of LUCAT1-WT (*p* < 0.001, Fig. 4A). And the miR-141-3p inhibitors enhanced this activity (*p* < 0.001, Fig. 4A). However, miR-141-3p mimics and miR-141-3p inhibitors had no effects on the luciferase activity of LUCAT1-MUT (Fig. 4A). Also, the correlation analysis showed that the LUCAT1 expression had a negative correlation with miR-141-3p (*r* = − 0.5846, *p* < 0.001, Fig. 4B). QBC939 and HuCCT1 cells were effectively transfected with si-LUCAT1 and miR-141-3p inhibitors using Lipofectamine 3000 (*p* < 0.001, Fig. 4C). Next, we explored the effects of miR-141-3p on the proliferation and migration of human cholangiocarcinoma cells QBC939 and HuCCT1. The growth conditions of QBC939 and HuCCT1 cells at 24, 48 and 72 h were detected by the CCK-8 method. miR-141-3p inhibitors reversed the proliferation- and migration-inhibitory effects of LUCAT1 silencing in both QBC939 and HuCCT1 cells (*p* < 0.001, Fig. 4D-F).

## Discussion

The majority of cholangiocarcinomas develop without any identifiable predisposing factors [27]. Consequently, the early diagnosis, treatment, and prognosis of cholangiocarcinoma remain significant challenges in clinical practice [28]. Currently, due to the challenges associated with obtaining pathologic and cytologic diagnoses, cholangiocarcinoma is primarily identified through imaging studies and clinical history [3]. Advanced cytologic techniques, such as fluorescence in situ hybridization, are known to improve diagnostic sensitivity. Thus, the search for independent biomarkers of CHOL has emerged as a key focus of current research. Studies have shown that abnormal lncRNA expression or function is closely linked to the development of various cancers [29]. Additionally, certain lncRNAs have been reported to influence cell proliferation, migration, and invasion in CHOL [30–32]. In this study, we investigated the clinical significance of lncRNA LUCAT1 in the diagnosis and prognosis of CHOL.

LUCAT1 has been identified in several cancers, including osteosarcoma, tongue squamous cell carcinoma, lung cancer, and ovarian cancer. Upregulated LUCAT1 expression has been shown to impact the prognosis of various tumors. Zhou et al. [33] reported that LUCAT1 is upregulated in colorectal cancer and is strongly associated with reduced overall survival in patients. Sun et al. [34] reported that LUCAT1 was up-regulated in non-small cell lung cancer, which was associated with poor prognosis. To further study the clinical value of LUCAT1 in CHOL, we conducted a five-year follow-up study involving 83 CHOL patients to assess the relation between LUCAT1 expression levels and patients’ prognosis. The ROC results showed that LUCAT1 could distinguish tumor tissue from normal tissue in CHOL patients. We observed that elevated LUCAT1 expression was strongly associated with poor CHOL prognosis Moreover, the tumor size, CA-19-9, and TNM-T-Stage were significantly correlated with the LUCAT1 expression. The Cox regression analysis indicated that LUCAT1 can be regarded as an independent prognostic indicator for CHOL. Additionally, Mou et al. [35] found that lncRNA LUCAT1 promotes breast cancer metastasis by regulating miR-5702. Wu et al. [36] found that LUCAT1 down-regulates MYC expression to promote colorectal cancer cell proliferation. Our findings confirmed that LUCAT1 expression is significantly upregulated in CHOL tumor tissues. Moreover, we found that the proliferation and migration of the human cholangiocarcinoma cell line QBC939 and HuCCT1 were suppressed by silencing LUCAT1. These findings suggest that LUCAT1 may serve as a potential biomarker for CHOL. miRNAs and lncRNAs are known to interact via the ceRNA mechanism [37]. There are mainly two main modes of action: one type is competitive binding of miRNA. lncRNA adsorbs miRNA through the reaction element (MRE) containing miRNA as a “sponge”. In this way, lncRNA can organize the binding of miRNA to mRNA, thereby relieving the inhibitory effect of miRNA on the target gene. Another one is to form a competitive regulatory axis of lncRNA-miRNA-mRNA. Our results showed that miR-141-3p mimics suppressed the luciferase activity of LUCAT1-WT, while miR-141-3p inhibitors enhanced it. However, they did not affect the luciferase activity of LUCAT1-MUT. Therefore, we hypothesize that LUCAT1 influences the progress of CHOL by regulating the expression of miR-141-3p. We successfully transfected miR-141-3p inhibitors into QBC939 and HuCCT1. Interestingly, miR-141-3p inhibitors reversed the inhibitory effect of silencing LUCAT1 on the proliferation and migration of QBC939 and HuCCT1 cells. These results indicated that LUCAT1 targets miR-141-3p to affect the proliferation and migration of cholangiocarcinoma cells.

## Conclusion

In conclusion, LUCAT1 is significantly upregulated in CHOL tissues and cell lines and is associated with poor prognosis. Functionally, it promotes tumor cell proliferation and migration by targeting miR-141-3p, identifying LUCAT1 as a promising diagnostic and prognostic biomarker in CHOL. However, further in vivo validation and multicenter clinical studies are warranted.

It should be pointed out that the samples in this study were mainly from The Central Hospital of Wuhan, Tongji Medical College, Huazhong University of Science and Technology, which has certain limitations in terms of sample diversity. Therefore, the generalizability of the conclusions needs to be further evaluated. In addition, in vitro cell experiments cannot mimic the complex microenvironment in vivo, which may affect the credibility of our mechanistic hypothesis. Therefore, this study needs to be further evaluated by clinical in vivo experiments.

## Data Availability

Corresponding authors may provide data and materials.
